# Supplementary material for: Separation of Coiled-Coil Structures in Lamin A/C Is Required for the Elongation of the Filament
Source: Cells. 2020 Dec 31;10(1):55. doi: 10.3390/cells10010055 (PMC7824274; doi:10.3390/cells10010055)
Supplement: Supplementary file 1 [file cells-10-00055-s001.pdf]

Supplementary Materials:

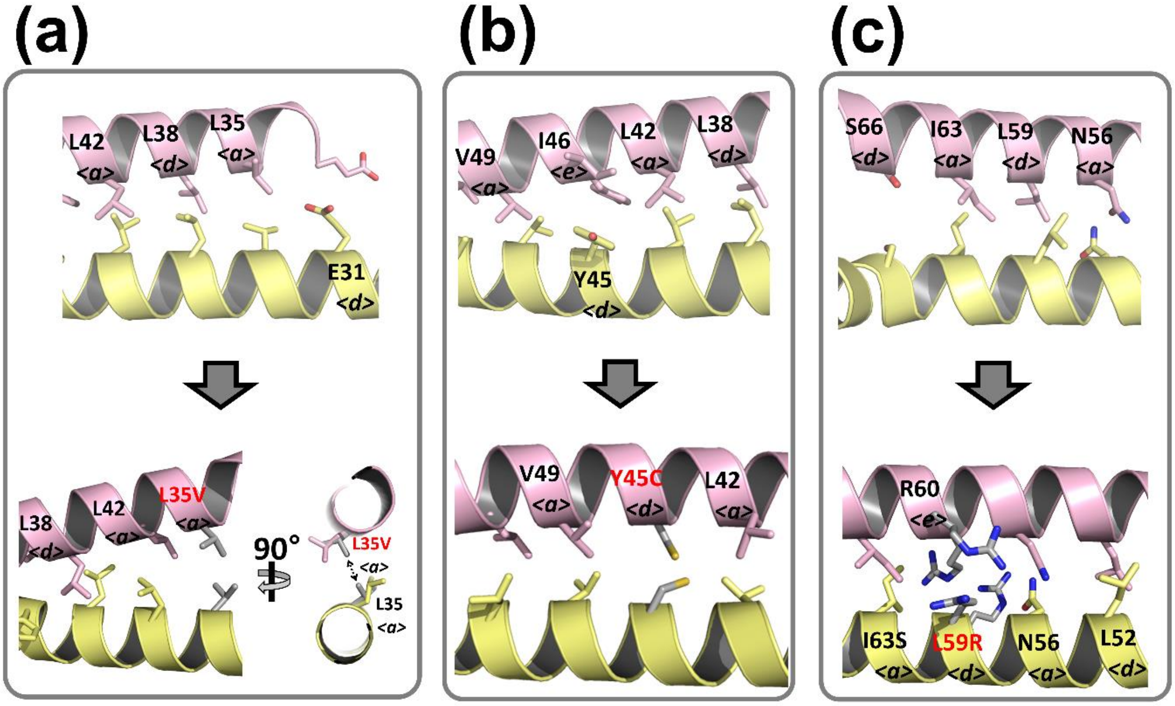

Supplementary Figure 1. The structural environment of L35V, Y45C, L59R, and I63S variants

The orthogonal views of the coiled-coil structure around (a) L35V, (b) Y45C, and (c) L59R and (d) I63S residues are presented. Each Tyr45 residue interacts with an Ile46 residue in the other protomer in the dimeric unit. Leu35, Leu59, and Ile63 residues adopt the ideal coiled-coil interaction. The predicted structural model of substituted residues (L35V, L45C, L59R, and I63S) is highlighted in red. The *a*, *d*, and *e* positions of the residues in the heptad repeat are labeled with an italic letter.

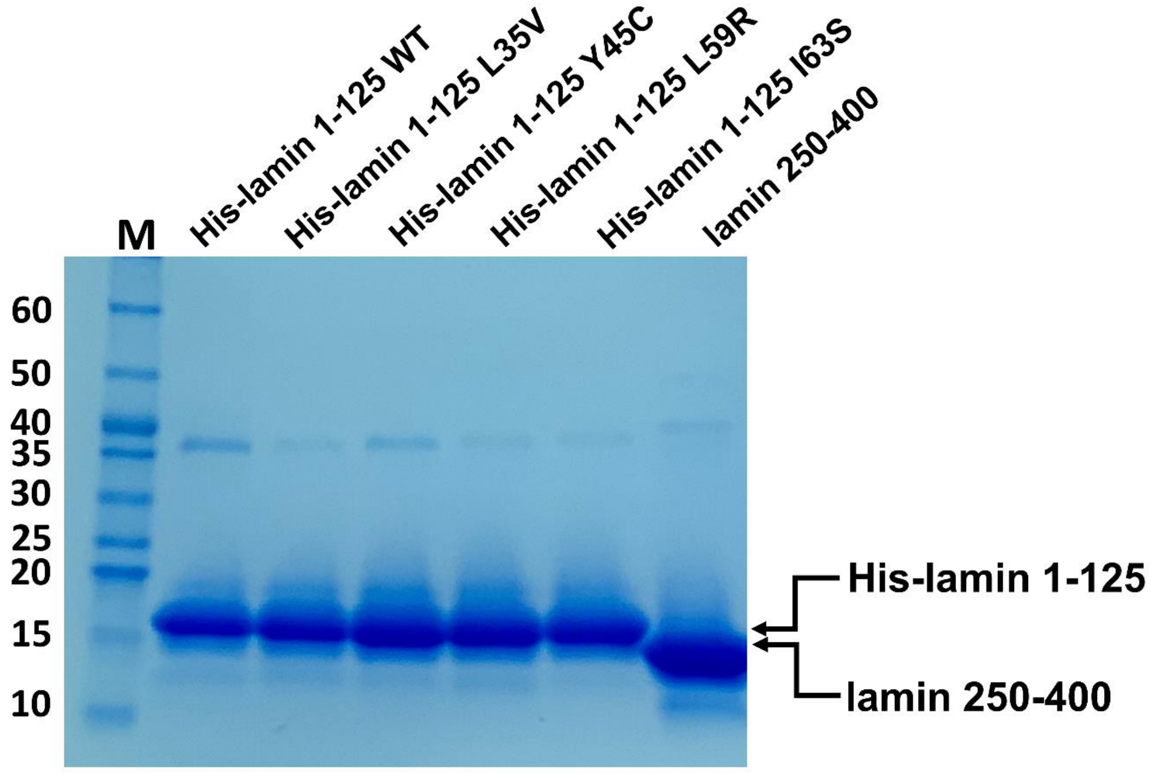

Supplementary Figure 2. The effects of L35V, Y45C, L59R, and I63S mutations on the binding strength between the His-tagged N-terminal fragment (residues 1-125) and the coil 2 fragment (residues 250-400) of lamin A/C

The used proteins for binding affinity in Fig. 2b.

(a)

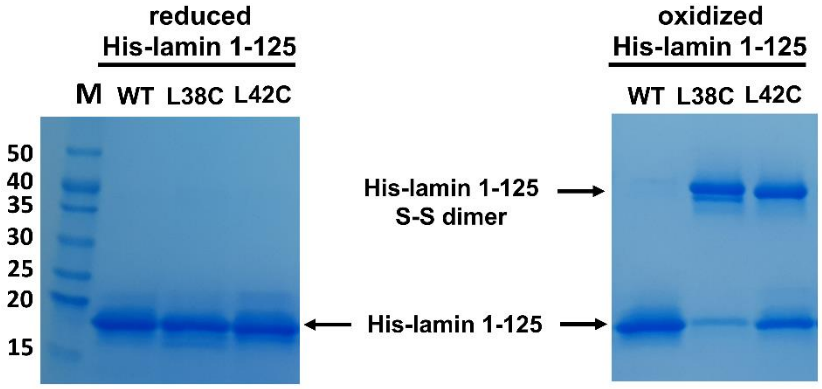

(b)

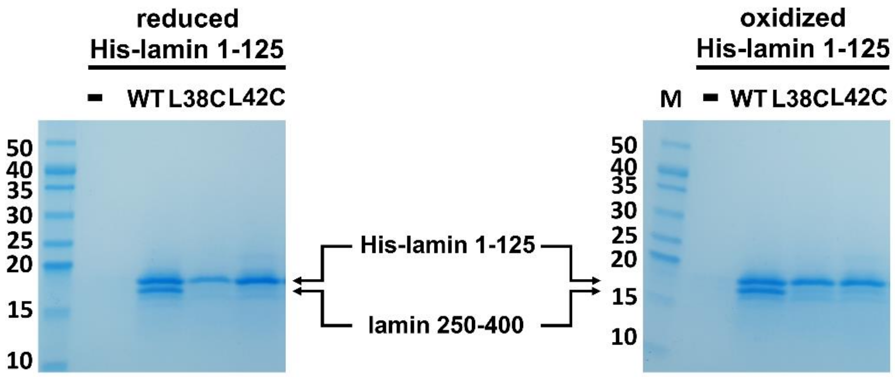

Supplementary Figure 3. The effect of the L38C and L42C mutations on the binding strength of the eA22 interaction

(a) Binding affinity between the lamin 250-400 (C-terminal of coil 2) and His-lamin 1-125 fragments (WT, L38C, and L42C) was analysed by pull-down assay. To analyse the effect of the disulfide bond, oxidized (*left*), and reduced (*right*) His-lamin 1-125 fragments were used for the pull-down assay. Lamin 250-400 fragments were incubated on empty (-) or His-lamin-bound (WT, L38C, and L42C) Ni-NTA resins. The used proteins are shown in (b).

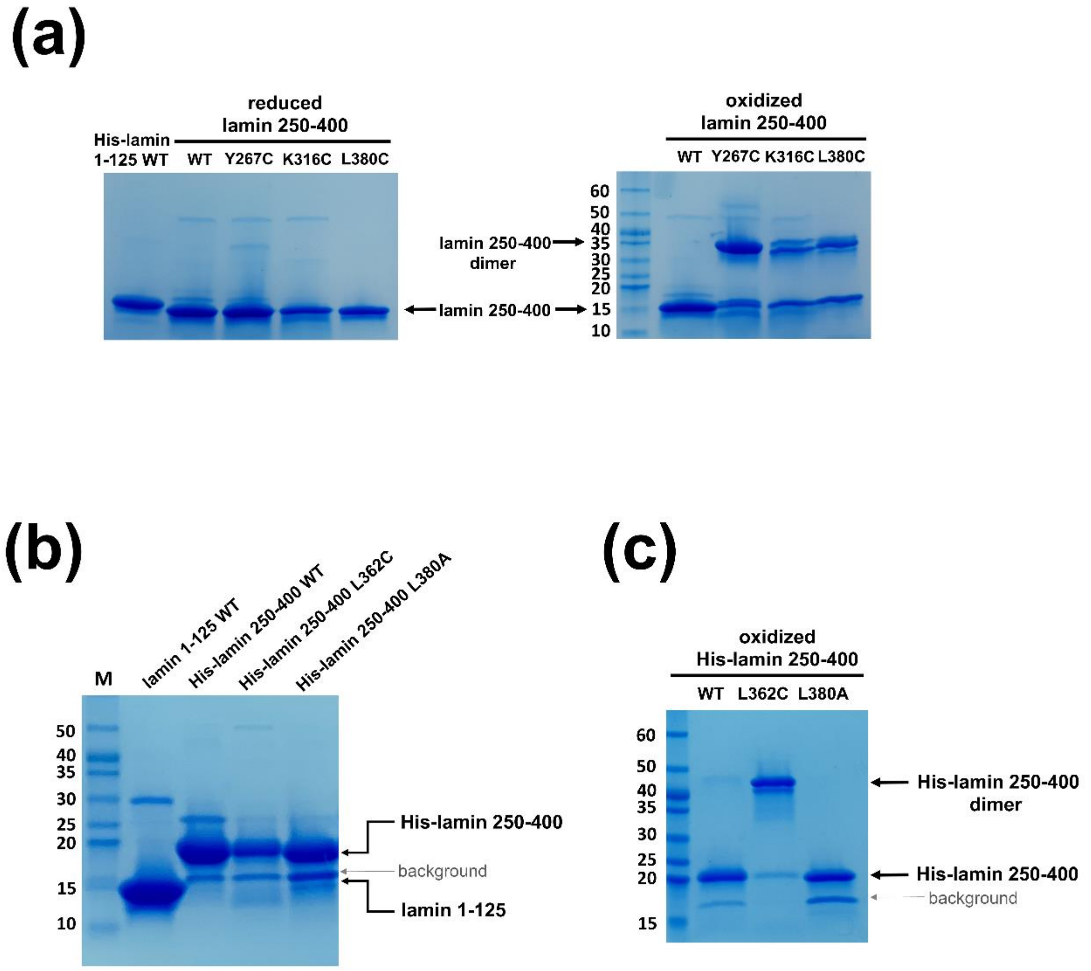

Supplementary Figure 4. Used proteins in the pull-down assays

(a), (b) The used proteins in the pull-down assay shown in Fig. 6 (a; Fig 6a, b; Fig 6b).

(c) The oxidized His-lamin 250-400 proteins in Fig. 6b identified by SDS-PAGE. Protein bands for the degraded His-lamin 250-400 fragments are seen between the molecular sizes of 15 and 20 kDa in SDS-PAGE, indicated by the grey arrow.

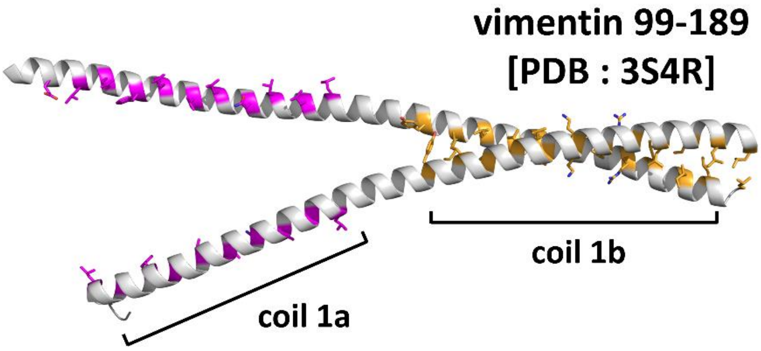

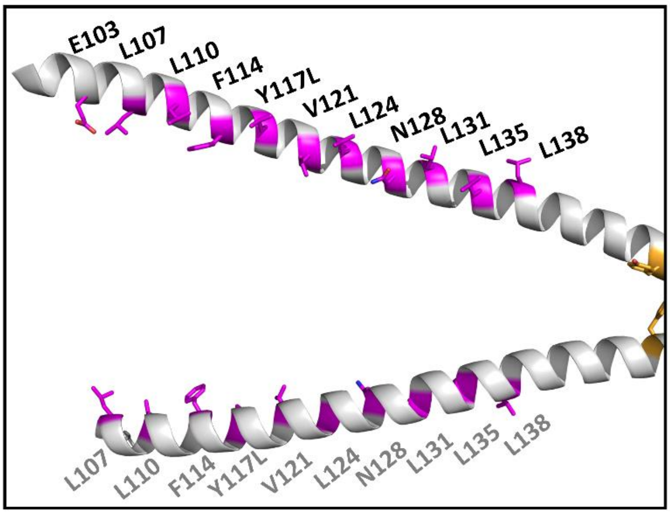

Supplementary Figure 5. Straightened and CC-separated conformations of coil 1a in vimentin (PDB 3S4R[1])

The ribbon diagram presents the N-terminal region composed of coil 1a, linker 1, and half of coil 1b. The coil 1a region is enlarged in the bottom box. The inter-helical hydrophobic residues of coil 1b are shown as yellow sticks. The residues in the *a* and *d* positions of the heptad repeat that do not form a coiled-coil dimer are colored magenta.

References

1. Nicolet, S., H. Herrmann, U. Aebi, and S. V. Strelkov. "Atomic Structure of Vimentin Coil 2." *J Struct Biol* 170, no. 2 (2010): 369–76.
